# Gamma irradiation exposure for collapsed cell junctions and reduced angiogenesis of 3-D in vitro blood vessels

**DOI:** 10.1038/s41598-021-97692-8

**Published:** 2021-09-14

**Authors:** Kyuhwan Na, Youngkyu Cho, Dong-Hee Choi, Myung-Jin Park, Ji-hun Yang, Seok Chung

**Affiliations:** 1 School of Mechanical Engineering, Korea University, Seoul, Republic of Korea; 2 Sensor lab, Smart Device Team, Samsung Research, Samsung Electronics Co. Ltd., Seoul R&D Campus, Seoul, Republic of Korea; 3 Division of Radiation Cancer Science, Korea Institute of Radiological and Medical Sciences, Seoul, Republic of Korea; 4 R&D Research Center, Next & Bio Inc., Seoul, Republic of Korea; 5 KU-KIST Graduate School of Converging Science, Korea University, Seoul, Republic of Korea

**Keywords:** Cell biology, Cell migration, Biotechnology, Tissue engineering

## Abstract

During radiotherapy, microenvironments neighboring the tumor are also exposed to gamma irradiation; this results in unexpected side effects. Blood vessels can serve as microenvironments for tumors and they play an important role in providing nutrients to tumors. This is mostly related to tumor progression, metastasis, and relapse after therapy. Many studies have been performed to obtain a better understanding of tumor vasculature after radiotherapy with in vitro models. However, compared to 3-D models, 2-D in vitro endothelial monolayers cannot physiologically reflect in vivo blood vessels. We previously remodeled the extracellular matrix (ECM) hydrogel that enhanced the tight barrier formation of 3-D blood vessels and the vascular endothelial growth factor (VEGF) gradient induced angiogenesis in a microfluidic device. In this study, the blood vessel model is further introduced to understand how gamma irradiation affects the endothelial monolayer. After the gamma irradiation exposure, we observed a collapsed endothelial barrier and a reduced angiogenic potential. Changes in the cell behaviors of the tip and stalk cells were also detected in the angiogenesis model after irradiation, which is difficult to observe in 2-D monolayer models. Therefore, the 3-D in vitro blood vessel model can be used to understand radiation-induced endothelial injuries.

## Introduction

Radiation treatment, which is also known as radiotherapy, has been widely applied to a variety of solid tumors in which it is used to kill the tumor cells directly or reduce the tumor mass^1–4^. There are several supportive cells in the tumor microenvironment and they can be damaged by radiation exposure, which can result in several side effects^5–8^. Blood vessels play an important role in supplying nutrients and oxygen to tumors; they also act as routes for other organs, which causes tumors to grow or become metastasized^9–13^. Indeed, as the tumor size increases, its metabolism is restricted owing to the limited diffusion of oxygen and nutrients. The tumor then recruits new blood vessels by secreting angiogenic activators such as vascular endothelial growth factor (VEGF), basic fibroblast growth factor (bFGF), and tumor necrosis factor alpha (TNF-a)^14–18^. Because these new blood vessels can affect the efficacy of in vivo radiotherapy for tumors, several kinds of anti-angiogenic drugs have been combined to suppress tumor regression effectively^19,20^.

Many researchers have reported that endothelial cells undergo changes in their molecular pathways and cell phenotypes after they are exposed to gamma irradiation^21–24^. In particular, the reorganization and reduction of cell–cell junctions between endothelial cells are major problems because they increase cell permeability and weaken barrier functions^25–27^. However, most of these studies were conducted on 2-D in vitro culture systems, in which the endothelial cells grow as a homogenous monolayer. As a result, they cannot reflect the physiological relevance of in vivo blood vessels. For instance, the morphology and biochemical signaling of the endothelial cells differ according to the cell–cell and cell–matrix interactions. In addition, soluble gradients such as nutrients, oxygen, and other stimuli are absent in 2-D culture systems^28–30^. Conversely, 3-D endothelial models have the potential to constitute more realistic biochemical and biomechanical microenvironments for blood vessels. These models can support diverse endothelial cell behaviors such as angiogenesis, vasculogenesis, and cell invasion.

In our previous research, we enhanced the permeability and barrier maintenance of a 3-D endothelial monolayer by remodeling the extracellular matrix (ECM) hydrogel. In addition, angiogenesis can be triggered by the VEGF gradient^31,32^. Because the majority of defects in irradiated endothelial cells result in a loss of barrier functions and cell motility, our 3-D endothelial cell model can be a powerful tool to understand the radiation damage to human blood vessels. This study demonstrates that gamma irradiation exposure weakens the barriers of the endothelial cell and it can be visualized through the diffusion of dextran molecules across the endothelial barriers. Furthermore, the genes that regulate the notch signaling pathway in angiogenesis, as well as the cell behaviors of the tip and stalk cells, change depending on the gamma irradiation doses. To the best of our knowledge, this is the first study to show how gamma irradiation exposure affects endothelial cell behaviors in the 3-D hydrogel-incorporated blood barrier model.

## Materials and methods

### Preparation of the microfluidic chip

The microfluidic channels were patterned by photolithography. First, the SU-8-100 photoresist (MicroChem, USA) was spin-coated onto a silicon wafer and then baked at 95 °C for 1 h. Using selective ultraviolet (UV) exposure, microfluidic channels were patterned on a silicon wafer. Polydimethylsiloxane (PDMS, SYLGARD 184, Dow, USA) was cured on this micropatterned silicon wafer with a conventional soft lithography procedure. Reservoirs were punched with 4 mm and 1 mm biopsy punches. The microfluidic chip was sterilized twice at 120 °C for 15 min and then dried at 80 °C overnight. The dried microfluidic chip and cover glass were bonded with an oxygen plasma treatment (FEMTOSCIENCE, Korea) for 1 min at 100 W. After the plasma treatment, the hydrophilic microchannels were filled with 1 mg/mL of PDL (poly-d-lysine, Sigma-Aldrich) hydrobromide solution to enhance the adhesion of the hydrogel to the surface of the PDMS and the cover glass. After incubating at 37 °C for 3 h, the microfluidic channels were washed twice with deionized distilled water (DW). The microfluidic chip was dried overnight at 80 °C and stored at room temperature before usage.

### Cell preparation

Primary human dermal microvascular endothelial cells (hMVEC; Lonza, cat. no. CC2546, Switzerland) were purchased and cultured in an EGM-2MV bullet kit (Lonza, cat. no. CC3202, Switzerland) at 37 °C in a humidified 5% CO_2_ incubator. The hMVECs were maintained until ~ 70% confluence in a 75T flask. In this study, the hMVECs in passage six were used for the experiments.

### ECM remodeling and cell seeding

Before seeding the cells, a pre-polymerized type I collagen (COL1) solution at a concentration of 2.0 mg/ml was prepared by dilution in a mixture of 10X phosphate buffered saline (PBS) and DW. In addition, the pH of the COL1 solution was adjusted with a 1.0 N NaOH solution. The COL1 solution was then poured into the four regions of the microfluidic chip. After pouring the COL1 solution, the chip was incubated at 37 °C for 30 min in a humidified chamber to prevent the hydrogel from drying. Subsequently, all the channels, except for the ECM channels, were filled with a cell culture medium, EGM-2MV. In a previously published paper from our laboratory, we showed that remodeling the ECM hydrogel results in a tight endothelial barrier in the microfluidic chip. Briefly, after the COL1 solution was poured into the gel regions and gelled, the cell culture channels were filled with serum-free culture medium. Then, growth factor reduced (GFR)-Matrigel solution (diluted in serum-free culture medium, 1:50) was added to the channels on ice for 5 min; this solution was coated onto the surface of the COL1 for more than 40 min in a 37 °C incubator. Subsequently, the hMVECs were harvested by trypsinization and seeded into the central channel of the microfluidic chip at a density of 2 × 10^4^/cm^2^ after the culture medium was removed from the side channels. The hMVECs were attached to the ECM by hydrostatic pressure for 2 h in a 37 °C incubator. After being cultured in the microfluidic channel for 4 days, they formed a monolayer. The medium was exchanged every day.

### Gamma irradiation treatment

Four days after cell seeding into the microfluidic chip, the hMVEC monolayers were exposed to gamma irradiation at 4 Gy and 8 Gy (BIOBEAM 8000, Gamma-Service Medical GmbH, Germany). After the gamma irradiation treatment, the hMVECs were cultured for more than 4 days. The medium was exchanged every day.

### Permeability analysis

To measure the permeability of the endothelial cell monolayer in the microfluidic chip, 10 µM of 40 kDa fluorescein isothiocyanate (FITC)–dextran solution was added to the central hMVECs channel and incubated for 3 h at 37 °C. The value of the permeability was calculated using Fick’s first law:

$$\begin{aligned} & J = - D\frac{\partial C}{{\partial x}}, \\ & {\text{Flux}} = - P\Delta C, \\ \end{aligned}$$

where $J$ is the flux, $D$ is the diffusion coefficient, $C$ is the concentration, $x$ is the position, and $P$ is the permeability of the endothelial monolayer. The diffusion coefficient of 40 kDa FITC-dextran in ECM hydrogel was assumed as 43 µm^2^/s with reference^33^. And, the diffusion profile of FITC–dextran was captured by fluorescence imaging, and the distribution was analyzed with the ImageJ software program (7.0a, GraphPad, www.graphpad.com).

### Immunofluorescence staining and quantification

The hMVECs in the microfluidic chip were fixed with 4% paraformaldehyde (PFA) at 25 °C for 30 min. Then, the cells were permeabilized with 0.1% Triton X-100 for 15 min at RT. In sequence, the cells were blocked with 3% bovine albumin serum (BSA) for 1 h at RT. Then, 50 µL of the diluted primary antibodies in the PBS solutions were added to the central channel and stored at 4 °C overnight. Secondary antibodies were conjugated with primary antibodies at RT for 1 h. All the primary and secondary antibodies are listed in Table 1. In each step, two sets of PBS washes were included. The fluorescence images were captured with a confocal laser-scanning microscope (LSM700; Carl Zeiss, Germany). Actin fibers were evaluated in total 45 randomly chosen hMVECs for each radiation doses and cells were divided into three groups differing in the number of actin stress fibers (0, 1–10, and > 10). The average intensity of VE-cadherin on cytoplasm and cell–cell border was measured by using Image J software program.

**Table 1:** Primary and secondary antibodies.

Marker	Cat.no. (company)	Dilution ratio
**Primary antibody**		
Anti-VE-cadherin	ab33168 (abcam)	1:200
Anti-Claudin 5	ab15106 (abcam)	1:200
Anti-DLL4	ab7280 (abcam)	1:1000
Anti-Cleaved Caspase 3	9664 (Cell signaling)	1:400
Anti-Cleaved Caspase 9	20750 (Cell signaling)	1:400
**Secondary antibody**		
Goat anti-rabbit IgG, Alexa fluor 488	A-11008 (Invitrogen)	1:200

### qRT-polymerase chain reaction (PCR) analysis

To extract the RNA from the hMVECs, the hMVECs in the microfluidic chip were harvested. Due to the number of hMVECs in one microfluidic chip was very small, hMVECs in about 5–10 microfluidic chips were collected as n number one (n = 2–3). Total RNA was isolated with the RNeasy Mini Kit (QIAGEN, Cat.no.74104, Germany) and the concentration of the total RNA was measured with a Nanodrop spectrophotometer (absorbance wavelength: 260 nm, ThermoFisher Scientific, Cat. no. ND-1000, USA). After the RNA normalization, cDNA was synthesized via reverse transcription with a high-capacity RNA-to-cDNA kit (Applied Biosystems, Cat.no. 4387406, USA) and StepOne Real-time PCR equipment (Applied Biosystems, Cat.no. Step-one plus, USA). We designed the PCR primers which are listed in Table 2. The PCR reactions were performed as duplicates using the StepOne system with the QuantiTect SYBR green PCR kit (QIAGEN, Cat.no. 204143, Germany). In addition, the gene expression of the target genes was normalized according to the housekeeping gene Gapdh and quantified with the comparative Ct method.

**Table 2:** Designed primers for qRT-PCR.

Gene	Forward	Reverse
GAPDH	TCCAGAACATCATCCCTGCC	GCCTGCTTCACCACCTTCTT
VE-cadherin	GCCAGTTCTTCCGAGTCACA	AACTCCGGGGCATTGTCATT
Claudin 5	GCGTGCTCTACCTGTTTTGC	GCTGAGTACTTCACGGGGAA
Notch1	GGCAACGTCAACACCTTGTC	CAACTGCCAGAACCTTGTGC
Jagged1	CTGACTCTTGCACTTCCCGT	CTACAACCGTGCCAGTGACT
DLL4	GtACATTGCCAGGGAGTGCT	CCACTTCGGCCACTATGTGT
Caspase 3	TGGGTGCTATTGTGAGGCGG	TGAGCAGGGCTCGCTAACTC
Caspase 9	CGGTGACGCAAGAGCGAATC	GATCAGCTGCCTGGCCTGAT

### Inducing the migration of the hMVECs in a microfluidic chip after gamma irradiation

The migration and angiogenesis of the hMVECs were induced by VEGF. In our microfluidic chip, a chemical gradient can be generated and maintained between the central and side channels. To induce the migration of the hMVECs, we introduced additional VEGF-A (PeproTech, Cat.no. 100-20, USA) with 50 ng/mL to the side medium channels daily. Due to our goal is to investigate how the gamma irradiation affects angiogenesis, additional VEGF-A was supplied to the side channels for 5 days after radiation treatment.

## Results

### Endothelial barrier formation in a 3-D microfluidic chip

**Figure 1:**
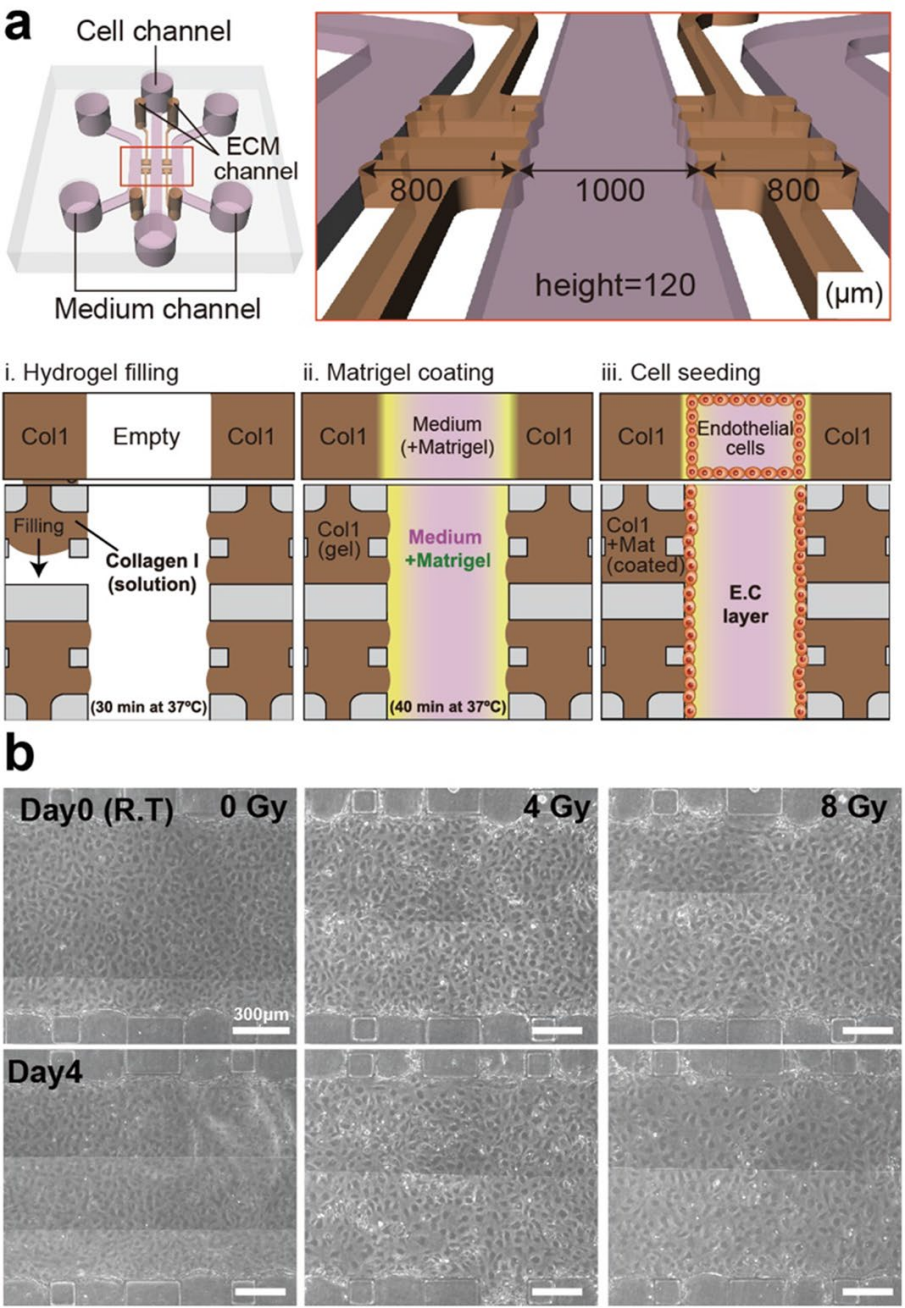
Schematic overview of endothelial barrier formation in our 3-D microfluidic chip and phase-contrast images of endothelial monolayer at day 0 and 4 according to irradiation doses (non-treated (0 Gy), 4 Gy, and 8 Gy). (**a**) Our microfluidic chip has three channels for seeding the cells and supplying the medium, and four regions for filling the hydrogels. After filling the hydrogels and remodeling the ECM according to our previous study, the hMVECs were cultured in central channels; they formed a monolayer within 4 days. (**b**) Phase-contrast images of the hMVECs monolayer in the microfluidic chip before and after the gamma irradiation treatment that was dose dependent. (Scale bar: 300 µm) Schematic and phase-contrast images were created with Adobe illustrator (CC 2019 23.0.1, www.adobe.com).

In our previous study, we fabricated a microfluidic chip for a 3-D cell culture that consisted of three parallel cell channels and four regions for hydrogel filling. In addition, we showed that Matrigel-coated type I collagen (BM-COL1) enhanced the barrier function of the endothelial cells with a high expression of tight and adhesion junctions^31^. In this study, we used this method to form a 3-D endothelial barrier, and hMVECs were used because this endothelial cell type displays superior barrier properties in comparison to umbilical vein endothelial cells (hUVECs)^34^. In our microfluidic chip, a hydrostatic pressure difference can be developed between the channels; this makes the cells more efficiently attached to BM-COL1. In addition, we determined that the hMVECs in the central channel proliferated and formed a confluent monolayer within 4 days after seeding the cells (Fig. 1a). To investigate how the gamma irradiation exposure affects the endothelial barrier, we exposed the monolayer of the hMVECs in the microfluidic chip to three doses (0 Gy for the control, 4 Gy, and 8 Gy) of gamma irradiation (Fig. 1b).

### Changes in the actin filament structures when subjected to gamma irradiation

**Figure 2:**
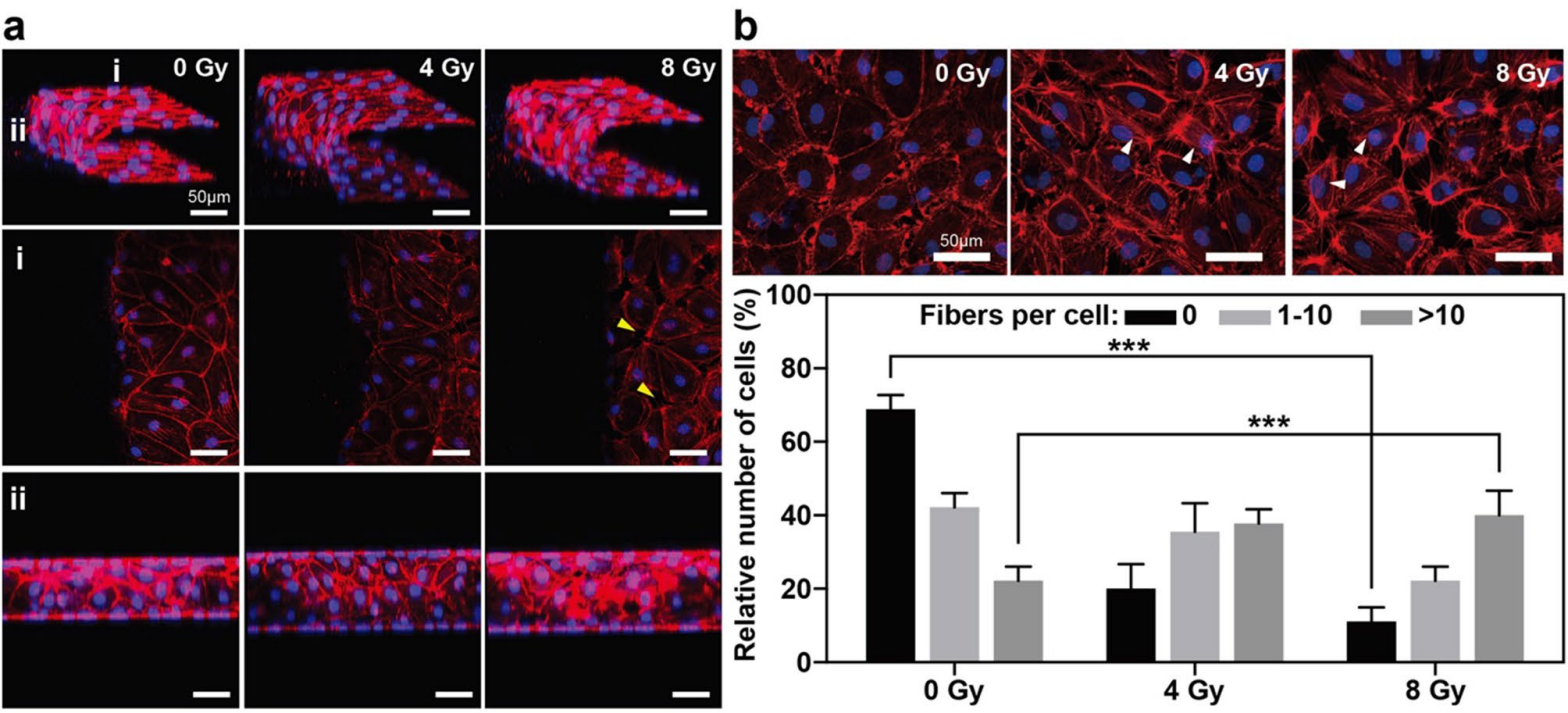
Immunofluorescence images of F-actin and quantification of actin stress fibers in hMVECs after gamma irradiation. (**a**) The structure and alignment of the actin filaments in the hMVECs changed after the gamma irradiation exposure. (**b**) Representative immunofluorescence images of F-actin and quantification for the percentage of cells with different numbers of actin stress fibers after gamma irradiation. (scale bar: 50 µm, *p < 0.05; **p < 0.01; ***p < 0.001, n = 3 (15 randomly chosen hMVECs = 1)). Statistical comparison were calculated with two-tailed t-tests using Prism (7.0a, GraphPad software, www.graphpad.com). Images were analyzed using ImageJ (1.52q, NIH, www.imagej.nih.gov/ij). All the images and graph were created with Adobe illustrator (CC 2019 23.0.1, www.adobe.com).

The actin filaments, which are also known as F-actin, are polymeric filaments that are composed of many globular actins (G-actins). They can determine the cell morphology and motility by actin polymerization. Likewise, these actin filaments in endothelial cells are important for controlling several kinds of cellular processes, which include cell–cell adhesion and cell migration^35–37^. Previous studies conducted with 2-D endothelial monolayer models suggested that the gamma irradiation treatment leads to endothelial cell injury as well as changes in the actin filament structures^22^. These changes in the actin filament structures were also observed in our 3-D endothelial barrier model. In the non-irradiated hMVEC monolayers, F-actin was locally expressed along the cell edge with a thin layer, and the actin bundles were clearly observed within the cytoplasm of the endothelial cells. However, 4 days after the gamma irradiation exposure, we can figure out that gamma irradiation exposure increases the proportion of hMVECs with a high content of stress fibers. In particular, for the hMVECs which were exposed to high dose (8 Gy) of gamma irradiation, significant changes in the proportion of cells with no stress fibers and over 10 stress fibers were observed (Fig. 2b, white arrows). In addition, it was also observed that the cell–cell contact was weakened (Fig. 2a, yellow arrows, Fig. S2).

### Gamma irradiation induced loss of cell-to-cell junction and increased endothelial barrier permeability

**Figure 3:**
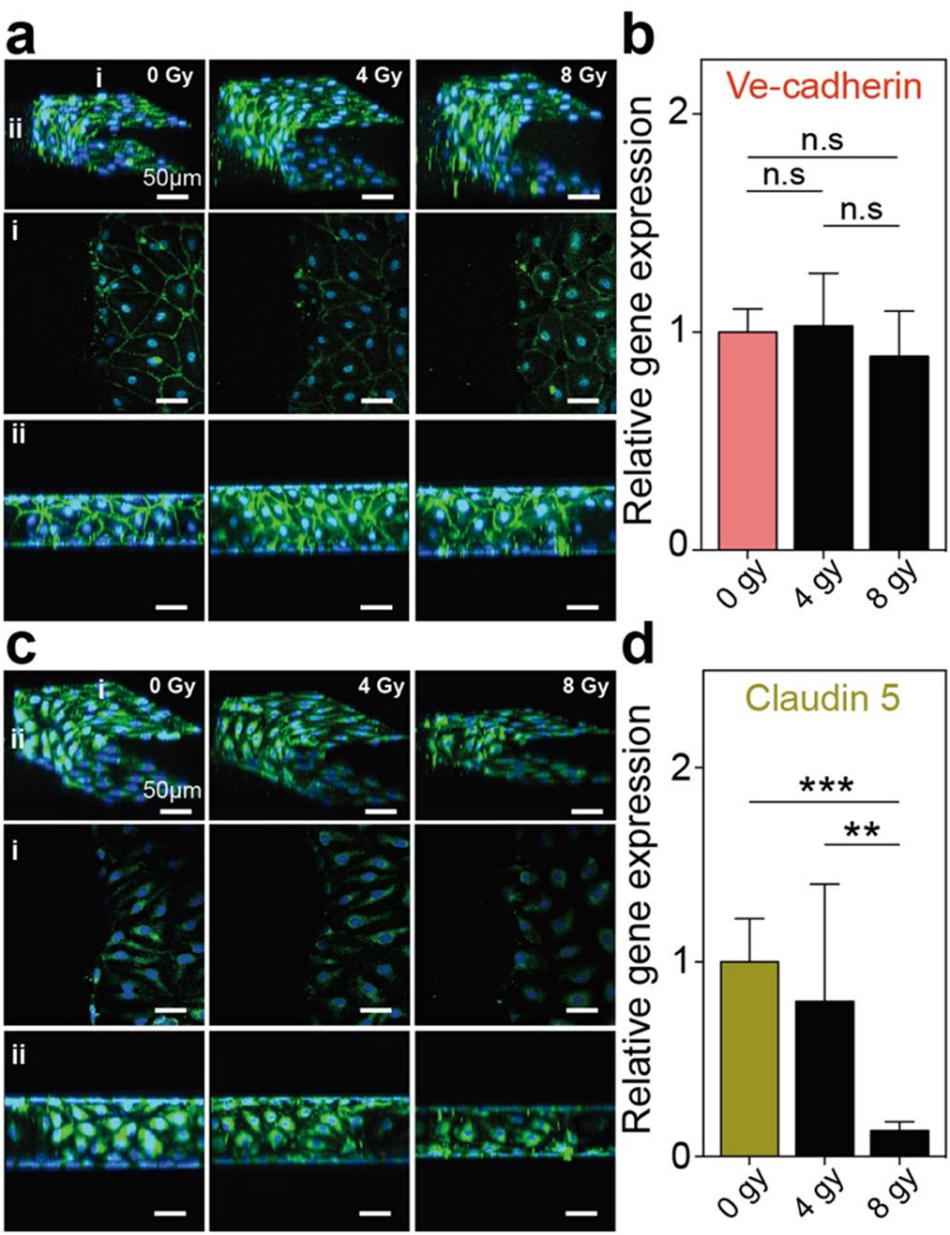
Immunofluorescence images and qRT-PCR for junction proteins (VE-cadherin and Claudin 5) showed delocalized and reduced expression after irradiation. Immunofluorescence images showed (**a**) delocalized VE-cadherin and (**c**) reduced Claudin 5 expression on the hMVECs. In addition, the relative gene expressions of (**b**) VE-cadherin and (**d**) Claudin 5 after the gamma irradiation exposure corresponded to the immunofluorescence images (scale bar: 50 µm; *p < 0.05; **p < 0.01; ***p < 0.001, n = 2–3). Statistical comparison were calculated with two-tailed t-tests using Prism (7.0a, GraphPad software, www.graphpad.com). Images and graphs were created with Adobe illustrator (CC 2019 23.0.1, www.adobe.com).

**Figure 4:**
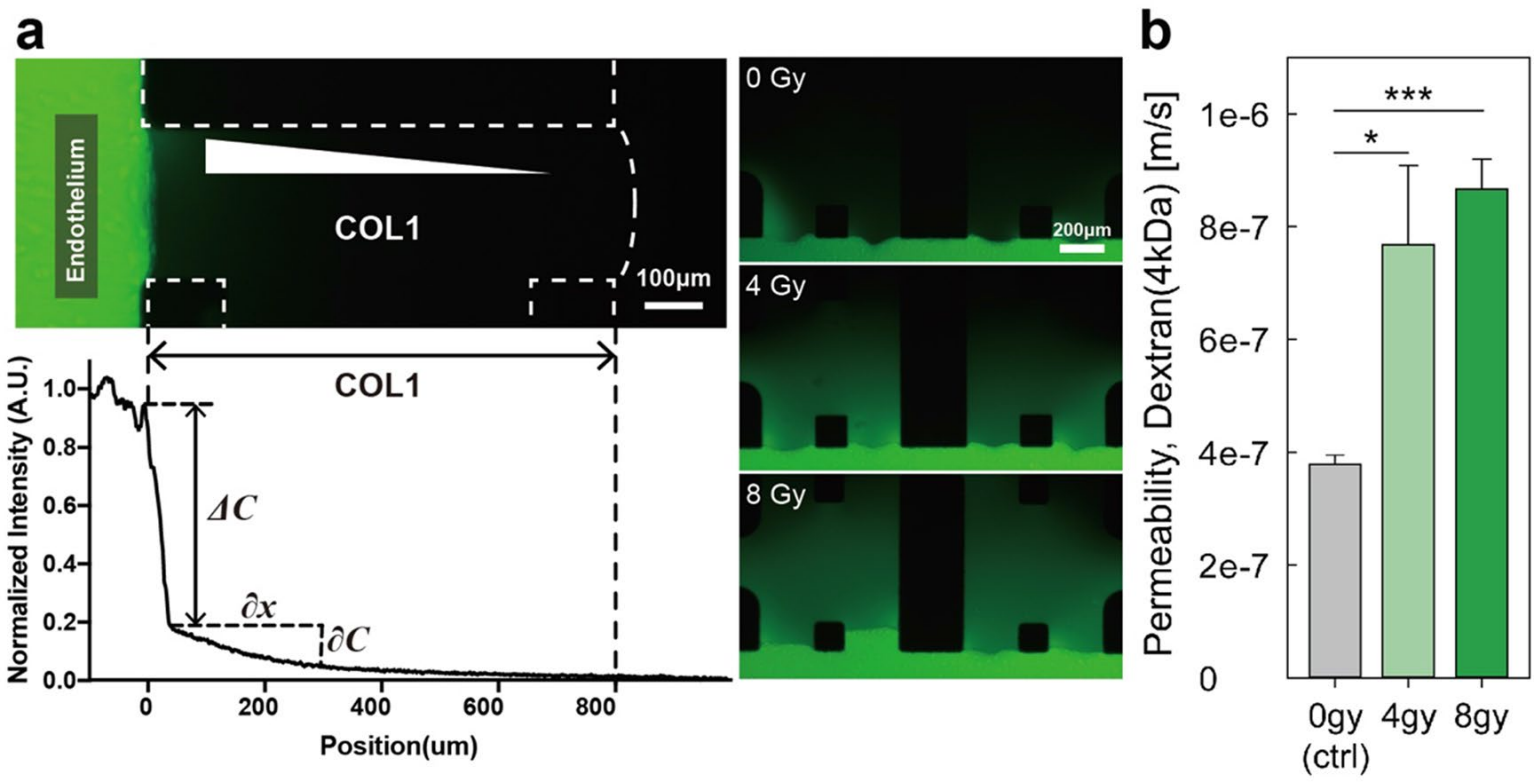
Representative fluorescence images and calculated permeability values of endothelial barriers after irradiation. (**a**) The tight hMVECs’ monolayer showed a sharp decrease in the intensity ($\Delta {\varvec{C}}$) at the interface between the hMVECs and the remodeled ECM. In addition, the intensity gradient $\left( {\frac{{\partial {\varvec{C}}}}{{\partial {\varvec{x}}}}} \right)$ in the ECM was measured in the normalized intensity profile with ImageJ (1.52q, NIH, www.imagej.nih.gov/ij). These intensity values vary according to the barrier permeability. (b) The permeability values of the hMVECs’ monolayer after 0, 4, and 8 Gy of gamma irradiation treatment (scale bar: 100 and 200 µm; *p < 0.05; **p < 0.01; ***p < 0.001, n = 4). Statistical comparison were calculated with two-tailed t-tests using Prism (7.0a, GraphPad software, www.graphpad.com). Images and graphs were created with Adobe illustrator (CC 2019 23.0.1, www.adobe.com).

In general, it is natural for endothelial cells to form a tight monolayer with the neighboring cells. There are several tight and adhesion junctions in endothelial cells, which include VE-cadherin, Claudin 5, Zo-1, and occludin. These tight junctions (TJs) and adhesion junctions (AJs) mediate the endothelial cell contacts, but they also provide different functions in the endothelial barrier. VE-cadherin, which is a unique AJ in endothelial cells, mainly contributes to the maintenance and stability of the endothelial cell–cell contacts. Conversely, TJs in the endothelium are mostly located along the apical region and they regulate the paracellular permeability of the ions or solutes across the endothelial cells. Among the many types of TJs, which include occludins, claudins, and junctional adhesion molecules (JAMs), Claudin 5 is the most abundant TJ protein in endothelial cells. Moreover, it can also control the intercellular barrier of the hMVECs^34,38^. In this study, we determined the alterations of these two junction proteins (VE-cadherin and Claudin 5) in the endothelial barrier after the gamma irradiation treatment. The non-irradiated hMVECs barrier revealed that VE-cadherin was localized at the cell–cell border, whereas the delocalized and scattered expression of VE-cadherin was discovered in the irradiated hMVECs (Figs. 3a and S3). Although the distribution of VE-cadherin in the hMVECs was changed according to the gamma irradiation exposure, there is little difference in the gene expression of VE-cadherin (Fig. 3b). This means that the gamma irradiation exposure induced the redistribution of VE-cadherin on the hMVECs; however, the quantity of the transcription mRNA was maintained even after the gamma irradiation. Conversely, the tight junction protein of the endothelial cells, Claudin 5, was considerably reduced depending on the gamma irradiation doses. Especially for the high dose (8 Gy) of the gamma irradiation exposure, these reduced expressions were significantly detected by qRT-PCR and the immunostaining analysis (Fig. 3c, d). Though the absolute quantity of transcript mRNA level primarily determines the protein amounts, the expression location of junction protein is also important for barrier function. Particularly, Claudin 5 was expressed at the cell–cell border in non-irradiated hMVECs whereas almost none of expressions at this region were observed in irradiated hMVECs (Fig. S4). Because Claudin 5 is a key protein that regulates the paracellular permeability of the endothelial barrier, we can infer that these delocalized Claudin 5 expression after gamma irradiation makes endothelial barrier more permeable. In effect, the paracellular permeability of the hydrophilic particles can be measured and monitored with 40 kDa fluorescein isothiocyanate (FITC)-dextran. FITC-dextrans significantly passed through the irradiated endothelial barrier for 3 h; the calculated permeability value was also increased up to two times more than the non-irradiated barrier (Fig. 4a, b). Due to radiation-induced endothelial cell apoptosis can be involved in the increase of permeability, we measured the gene expression levels of Caspase 3/9 which are biomarkers for cell apoptosis. Though a slight increase in the expression level for Caspase 3/9 was observed after irradiation, but there was no statistically significant difference between non- and irradiated hMVECs. However, by immunofluorescence staining, the number of apoptotic hMVECs was increased up to double after irradiation treatment (Fig. S1). Furthermore, the shrunken cytoplasm which is one of typical morphological changes in cell apoptosis was also observed in apoptotic hMVECs (Fig. S1, yellow dotted circle). Because small defects affect the whole endothelium integrity and determine barrier permeability, we can suppose that the irradiation induced hMVECs apoptosis provokes the collapse of endothelial barrier. Put together, we concluded that both the decreased tight junction protein Claudin 5 and hMVECs apoptosis can be major causes for defective hMVECs barrier function.

### Monitoring the cell migration of the hMVECs in the microfluidic after gamma irradiation

The main advantage of our 3-D blood vessel model is that it can develop well-defined chemical gradients and monitor the cellular behaviors in the 3-D ECM hydrogel. Our previous research demonstrated that the VEGF gradient attracted endothelial cells into the ECM, which mimics in vivo angiogenic sprouting^32^. In tumor microenvironments, tumors secrete a variety of growth factors in order to recruit new blood vessels (angiogenesis), which can determine the efficacy of tumor chemotherapy and radiotherapy. As a result, recent tumor therapies have targeted the removal of tumors and inhibited tumor vasculature.

**Figure 5:**
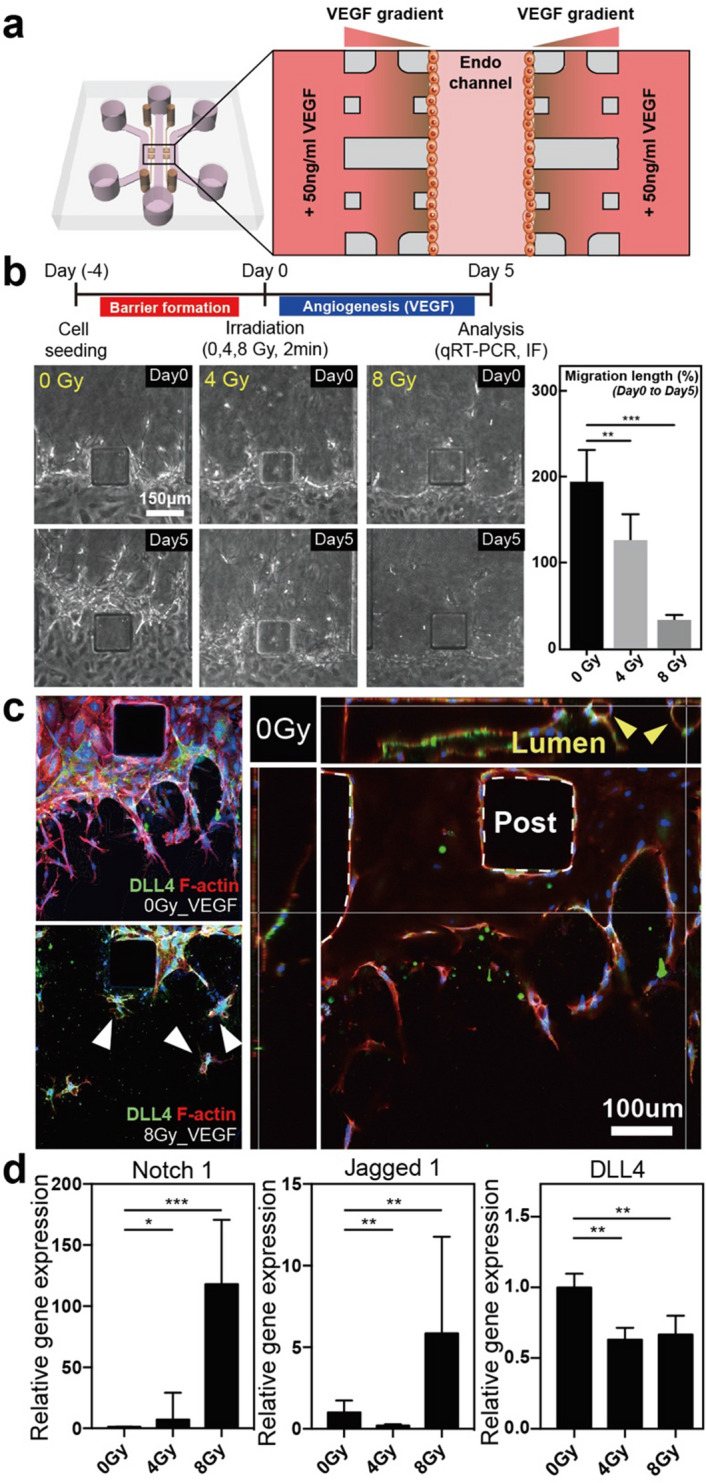
Schematic overview for inducing angiogenesis with VEGF gradient and defective angiogenesis in gamma-irradiated endothelial monolayer (**a**) By adding an additional 50 ng/mL of VEGF-A into the side channels, a gradient of VEGF-A was generated between the side channels and the central channel. (**b**) After the gamma irradiation treatment, endothelial monolayers were induced to angiogenesis for 5 more days. The endothelial monolayer that was exposed to high-dose (8 Gy) gamma irradiation showed a reduced cell migration into the hydrogel in comparison to the non-irradiated one. (n = 3) (**c**) In addition, the immunofluorescence images showed disrupted lumen structures in the gamma-irradiated endothelial monolayer. (**d**) Relative gene expressions for Notch1, Jagged1, and Dll4 showed large gaps among the non- and irradiated endothelial cells (scale bar: 100 and 150 µm; *p < 0.05; **p < 0.01; ***p < 0.001, n = 2–3). Statistical comparison were calculated with two-tailed t-tests using Prism (7.0a, GraphPad software, www.graphpad.com). Adobe illustrator (CC 2019 23.0.1, www.adobe.com) was used for creating schematic, phase-contrast images, immunofluorescence images and graphs.

To study the effect of gamma irradiation on endothelial angiogenesis, we utilized 3-D blood vessel barrier models as shown in “Endothelial barrier formation in a 3-D microfluidic chip” section. VEGF-A, which is a cytokine that attracts endothelial cells, was introduced to the side channels for an additional 5 days to develop the VEGF-A gradient between the central and side channels (Fig. 5a). We can observe that hMVECs with VEGF-A gradient migrated into the ECM hydrogel while almost none of cells were migrated without VEGF-A gradient. And, these VEGF-A induced cell migrations were also observed even after radiation exposure (Fig. S5). Although endothelial sensitivity to gamma irradiation differs according to the origin and types of endothelial cells, microvascular endothelial cells used in this paper are known to be radio-sensitive endothelial cell types^39^. Previous studies have demonstrated that gamma irradiation exposure suppresses angiogenesis in normal endothelial cells^40^. In our model, we also determined that the migration of hMVECs into the ECM hydrogel was suppressed in irradiated endothelial cells under a high-dose (8 Gy) irradiation (Fig. 5b). Moreover, the sprout morphology was clearly distinguished between non-irradiated and irradiated endothelial cells. Non-irradiated hMVECs actively migrated into the ECM hydrogel along the VEGF gradient and formed lumen structures. By contrast, the irradiated hMVECs revealed DLL4^+^ single cells that are scattered in the ECM discontinuously (Fig. 5c).

At this point, there are two factors that lead to abnormal sprouting. First, we previously confirmed that gamma irradiation exposure reduced the cell–cell junctions. The collective migration of the tip cells and the proliferative extension of the stalk cells are required in sprouting angiogenesis; they are largely affected by the cell junctions^41,42^. Thus, the weakened cell–cell junctions between the irradiated tip and the stalk cells can lead to the disruption of the stable integrity of these two cells. The next possible factor for suppressed angiogenesis in the irradiated hMVECs is the changes in the gene expression that regulate the cell behaviors and processes. By performing the qRT-PCR analysis, we can observe that the gene expressions of Notch1 and Jagged1 were up-regulated with high-dose (8 Gy) irradiation treated hMVECs (Fig. 5d). By contrast, the expression of Dll4 slightly decreased after the irradiation in comparison to the non-irradiated hMVECs.

It has been reported that Notch1 increases significantly with gamma irradiation and the notch signaling pathway is highly related to angiogenesis and the maturation of blood vessels^43,44^. Notch1 is regulated by its ligands Dll4 and Jagged1, which have opposing roles in angiogenesis. VEGF–VEGF receptor2 (VEGFR2) signaling, which is a key initiator of angiogenesis, induces Dll4 expression in the tip cells. In turn, Dll4 upregulates Notch1 expression and Dll4/Notch signaling reduces VEGFR2 expression in the neighboring stalk cells by inhibiting the tip cell selection. By contrast, Jagged1/Notch signaling acts as an antagonist to Dll4/Notch signaling by promoting the tip cell selection and sprouting. Likewise, the balance between these two notch signaling pathways is important for proper sprouting angiogenesis. However, the abnormally increased expression of Notch1 due to gamma irradiation exposure can lead to the dysfunction of endothelial cells to regulate the balance between tip and stalk cells, even under the VEGF stimuli.

Recent studies have proposed that the activation of Notch1 leads to the stabilization of the vessels by inhibiting the endothelial cell proliferation^45^. This means that the overexpression of Notch1 could inhibit the proliferation and reduce the filopodia extension of the stalk cells. In other words, although similar levels of tip cells migrated into the ECM hydrogel after the gamma irradiation exposure, the stalk cells cannot follow up the tip cells and form lumen structures (Fig. 5b, c). Of course, this defective angiogenesis can occur for the overexpression of Notch1 in irradiated endothelial cells as well as cell senescence due to DNA damage.

## Conclusion

In this study, we proposed a 3-D blood vessel model for studying the radiation-induced defects in endothelial barrier functions and the angiogenic potential. Because blood vessels play a major role in supplying nutrients to tumors and determine tumor growth, or even regression after tumor therapy, many kinds of in vitro endothelial models have been used. However, little is known about the cellular behaviors of endothelial cells because of the limitations of conventional 2-D endothelial cell models, in which the interactions of the cell–cell or cell–extracellular matrix cannot be represented. As a result, the native characteristics of the blood vessel can be lost despite the convenience of the model. To overcome these drawbacks in the 2-D endothelial monolayer models, we introduced a 3-D blood vessel barrier model in a microfluidic chip. In our chip, the endothelial cells formed a tight and stable monolayer by remodeling the ECM hydrogel. After gamma irradiation (4 and 8 Gy), the number of apoptotic hMVECs was increased and the fiber structure of the hMVECs was reorganized. In addition, the reduction of tight junction protein Claudin 5 in irradiated hMVECs was significantly observed. These small defects in endothelial cells can affect the whole endothelium integrity and largely related to the increasing molecular permeability after gamma irradiation.

Furthermore, during tumor progression or regression after radiotherapy, tumors secrete growth factors or cytokines to recruit new blood vessels in a process called angiogenesis. However, in 2-D cell culture systems, it is difficult to apply the gradient of chemokines; in our 3-D microfluidic system, gradients can be maintained. In angiogenesis, the tip and stalk cells showed different cell phenotypes; the systematic serial migration of these cells is important for forming tight lumen structures during angiogenesis. However, it is difficult to distinguish the tip and stalk cells in 2-D monolayer models because these cells have a homogeneous phenotype. By contrast, these cell-specific behaviors can be easily observed in our 3-D vessel model. In effect, although several studies have reported that gamma irradiation exposure can suppress angiogenesis by showing changes in the gene expression, protein level, or coverage area, little is known about the changes in the behaviors of the tip and stalk cells. However, we can see that the migratory tip cells were reduced slightly after gamma irradiation. In addition, the major defect is that the stalk cells cannot follow the tip cells, and the endothelial cells cannot form tubes. This can be effectively visualized in terms of how the gamma irradiation affects the barrier functions and the angiogenesis of the endothelial cells. Therefore, we propose that our 3-D endothelial models can be applied to understand further the radiation-induced vascular changes by observing single endothelial cell behaviors within the ECM hydrogel. In addition, these advanced 3-D vessel models can be expanded to incorporate tumor cells or spheroids to investigate the interaction between the tumor and blood vessels after gamma irradiation exposure.

## Supplementary Information

Supplementary Figures.
