# Supplementary material for: Gamma irradiation exposure for collapsed cell junctions and reduced angiogenesis of 3-D in vitro blood vessels
Source: Sci Rep. 2021 Sep 14;11:18230. doi: 10.1038/s41598-021-97692-8 (PMC8440565; doi:10.1038/s41598-021-97692-8)
Supplement: Supplementary file 1 — Supplementary Figures. [file 41598_2021_97692_MOESM1_ESM.docx]

**Supplementary Information**


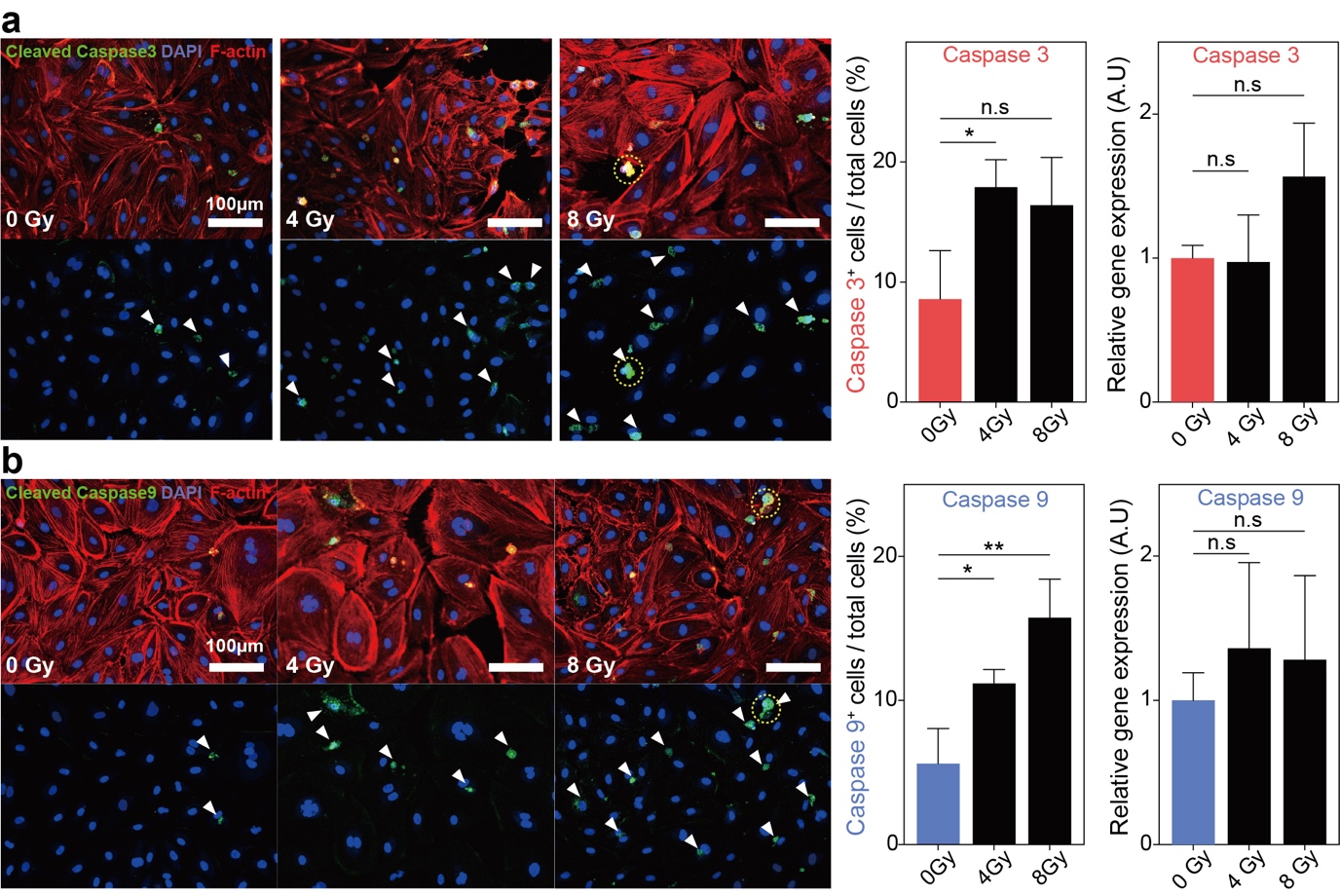


Figure S1. Immunofluorescence (IF) staining and qRT-PCR analysis for cell apoptosis markers (a) Caspase 3 and (b) Caspase 9 after radiation exposure.
By immunofluorescence staining, Caspase 3-positive and Caspase 9-positive hMVECs were observed up to double after radiation exposure compared to non-irradiated hMVECs (n=3). Also, the levels of mRNA for Caspase 3/9 were slightly increased even though there is no statistically significant difference (n=2, normalized to Gapdh). Statistical comparison were calculated with two-tailed t-tests using Prism (7.0a, GraphPad software, [www.graphpad.com](http://www.graphpad.com)) and graphs were created with Adobe illustrator (CC 2019 23.0.1, [www.adobe.com](http://www.adobe.com))


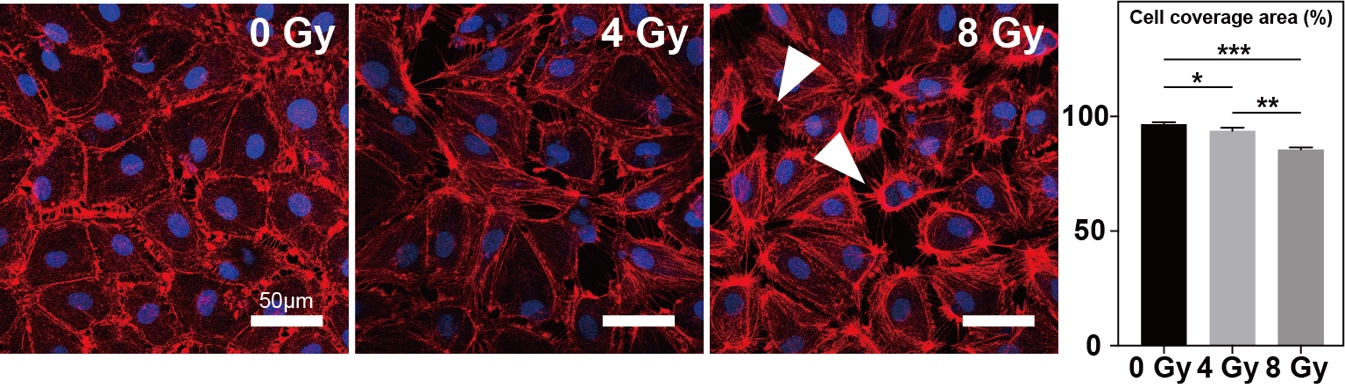


Figure S2. Decreased in cell coverage area after irradiation exposure.
Non-irradiated hMVECs monolayer covers over 95% of the surface whereas only 85% of cell surface was covered with 8 Gy irradiated hMVECs.
 (scale bar: 50µm, * p < 0.05; ** p < 0.01; *** p < 0.001, n=3)
Statistical comparison were calculated with two-tailed t-tests using Prism (7.0a, GraphPad software, [www.graphpad.com](http://www.graphpad.com)). Images and graphs were created with Adobe illustrator (CC 2019 23.0.1, [www.adobe.com](http://www.adobe.com))


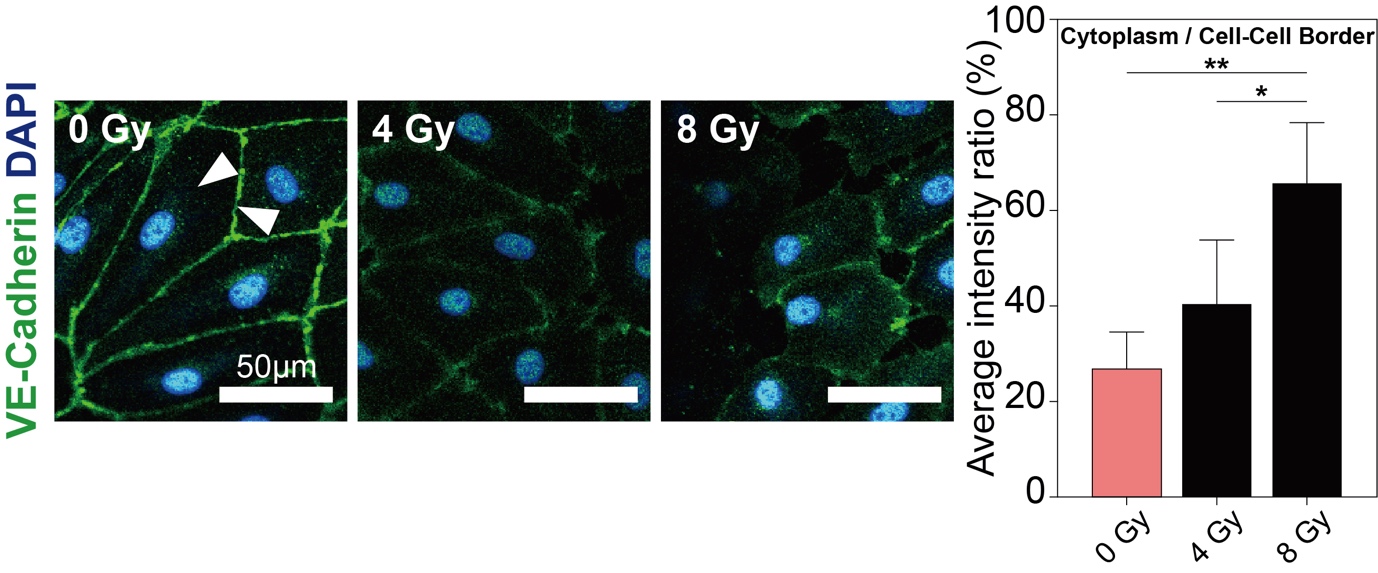


Figure S3. Representative immunofluorescence images of VE-cadherin on non- /irradiated-hMVECs and graph of average intensity ratio of its expression at cytoplasm to cell-cell border. VE-cadherin was strongly expressed at the cell-cell border in non-irradiated hMVECs (white arrows) whereas these localized VE-cadherin expressions were degraded and delocalized with irradiation (scale bar: 50µm, * p < 0.05; ** p < 0.01; *** p < 0.001, n=4)
Statistical comparison were calculated with two-tailed t-tests using Prism (7.0a, GraphPad software, [www.graphpad.com](http://www.graphpad.com)). Images and graphs were created with Adobe illustrator (CC 2019 23.0.1, [www.adobe.com](http://www.adobe.com))

**
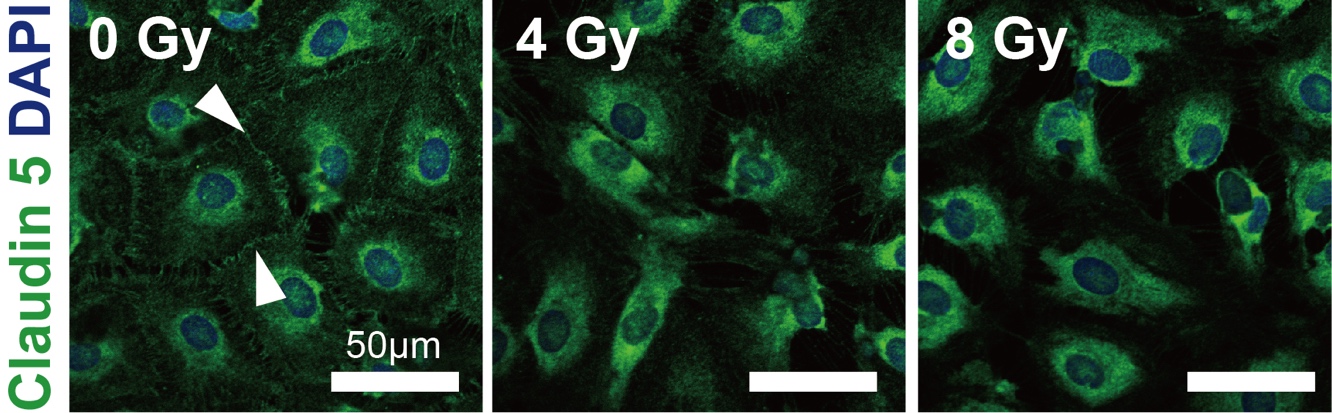
**

Figure S4. Immunofluorescence images of Claudin 5 on non- and irradiated hMVECs. Claudin 5 was expressed at the cell-cell border in non-irradated hMVECs (white arrows) whereas these localized Claudin 5 expressions were not observed in irradiated-hMVECs (scale bar: 50µm)
Images were created with Adobe illustrator (CC 2019 23.0.1, [www.adobe.com](http://www.adobe.com))


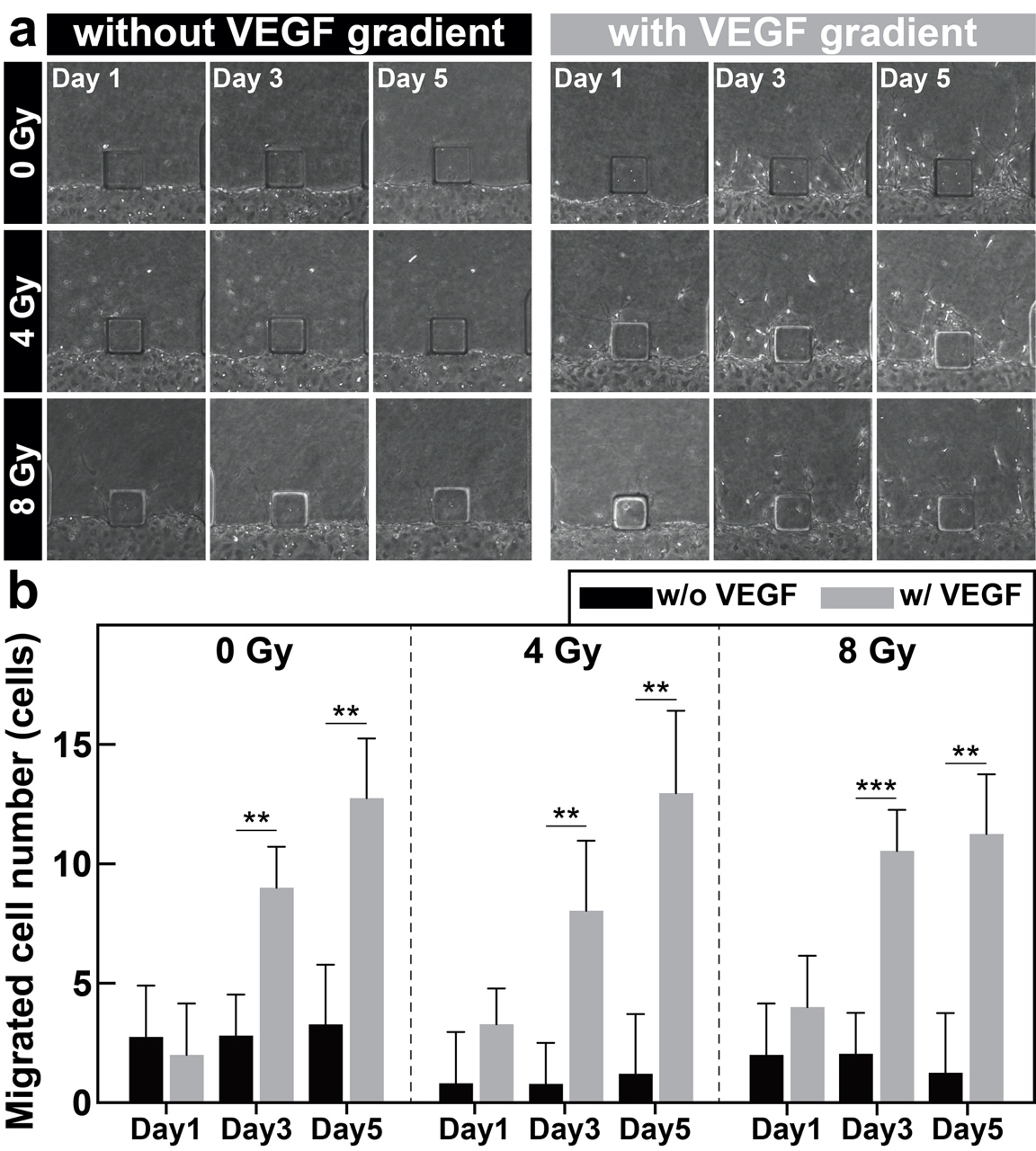


Figure S5. VEGF gradient induced hMVECs migration into ECM hydrogel. (a) Phase-contrast images of both non- and irradiated-hMVECs migration with or without VEGF gradient. (b) Graph of the number of migrated cells according to the presence of VEGF gradient and radiation exposure
(scale bar: 200µm, * p < 0.05; ** p < 0.01; *** p < 0.001, n=4)
Statistical comparison were calculated with two-tailed t-tests using Prism (7.0a, GraphPad software, [www.graphpad.com](http://www.graphpad.com)). Images and graphs were created with Adobe illustrator (CC 2019 23.0.1, [www.adobe.com](http://www.adobe.com))
